# Secondary vancomycin prophylaxis during antibiotic re-exposure for the prevention of recurrent *Clostridioides difficile* infections: a systematic review and Bayesian meta-analysis

**DOI:** 10.1093/jacamr/dlag141

**Published:** 2026-07-23

**Authors:** Connor Prosty, Mark Sorin, Emily G McDonald, Todd C Lee

**Affiliations:** Faculty of Medicine, McGill University, Montréal, Québec, Canada; Division of Experimental Medicine, Department of Medicine, McGill University, Montréal, Québec, Canada; Faculty of Medicine, McGill University, Montréal, Québec, Canada; Division of Experimental Medicine, Department of Medicine, McGill University, Montréal, Québec, Canada; Clinical Practice Assessment Unit, Department of Medicine, McGill University Health Centre, Montréal, Québec, Canada; Division of General Internal Medicine, Department of Medicine, McGill University Health Centre, Montréal, Québec, Canada; Division of Experimental Medicine, Department of Medicine, McGill University, Montréal, Québec, Canada; Clinical Practice Assessment Unit, Department of Medicine, McGill University Health Centre, Montréal, Québec, Canada; Division of Infectious Diseases, Department of Medicine, McGill University Health Centre, Montréal, Québec, Canada

## Abstract

**Background:**

Antibiotic re-exposure after an episode of *Clostridioides difficile* infection (CDI) is common and is a strong risk factor for recurrent CDI (rCDI). Two randomized controlled trials (RCTs) of vancomycin prophylaxis during antibiotic re-exposure were recently published, which we sought to evaluate by meta-analysis.

**Methods:**

We updated a recent systematic review search of MEDLINE and Embase via Ovid to 9 March 2026. RCTs of vancomycin during antibiotic re-exposure as secondary CDI prophylaxis were included. Quality was assessed by the Cochrane RoB2 tool. A Bayesian meta-analysis of 56-day rCDI was conducted on the odds ratio (OR) scale using a weakly informative prior. The probabilities of any benefit (i.e. OR < 1) and that the number needed to treat (NNT) was <20 (i.e. risk difference > 5%) were computed from the posterior. The 95% predictive interval (95% PI) was computed, which corresponds to the predicted range of the effect estimate of a future RCT.

**Results:**

Two RCTs at low risk of bias were included, which comprised 102 patients. The pooled OR was 0.61 [95% credible interval (95% CrI) = 0.27–1.40], which corresponded to a probability of any benefit with vancomycin prophylaxis of 88.0% and a probability that the NNT was <20 of 75.9%. The 95% PI was 0.19–2.00.

**Conclusions:**

Based on two small RCTs, there is a reasonable probability that secondary vancomycin prophylaxis during antibiotic re-exposure reduces 56-day rCDI. However, these results remain uncertain as the 95% PI is wide and consequently larger trials are needed.

## Introduction

Recurrent *Clostridioides difficile* infections (rCDI) are important causes of morbidity and healthcare cost.^1^ One of the strongest and most common risk factors for rCDI is re-exposure to systemic antibiotics.^2^ Secondary vancomycin prophylaxis during antibiotic re-exposure has been proposed as a potential strategy to mitigate this risk; however, the evidence base informing this practice was, until recently, solely observational studies at risk of bias.^3^ As a result, the Infectious Diseases Society of America’s guidelines have refrained from making recommendations on secondary vancomycin prophylaxis^4^ and global clinical practice varies widely.^5^ With the publication of two recent randomized clinical trials (RCTs),^6,7^ we sought to provide more objective meta-analytic estimates on the efficacy of secondary vancomycin prophylaxis.

## Methods

### Protocol

A pre-specified protocol was published on PROSPERO (CRD420261336400) and complies with guidance from PRISMA^8^ and Cochrane.^9^

### Search strategy

We updated a search strategy of Embase, MEDLINE, and CENTRAL from our recent systematic review from 24 May 2024 (end date of the prior search) to 9 March 2026.^3^ Grey literature, conference abstracts, and paediatric and animal studies were removed, and results were filtered with an automated RCT classifier.^10^

### Study selection

Retained articles were screened first through title and abstract and then full-text by two independent reviewers. RCTs of secondary vancomycin prophylaxis for the prevention of rCDI in adult patients re-exposed to systemic antibiotics were included. Observational, primary prophylaxis, and paediatric-only studies were excluded. Disagreements were resolved by consensus.

### Data extraction

Using a pre-specified form, data were extracted in duplicate by independent reviewers and discrepancies were resolved by consensus. The following variables were extracted: country, inclusion and exclusion criteria, arms, rCDI definition, and 56-day (±4 days) rCDI.^4^

### Quality assessment

Study quality was assessed in duplicate by independent reviewers using the Cochrane Risk of Bias 2 tool.^11^ The certainty of evidence was evaluated using Grading of Recommendations Assessment, Development, and Evaluation (GRADE).^12^

### Statistical analyses

56-day rCDI was pooled by Bayesian random-effects meta-analysis on the odds ratio (OR) scale with a 95% credible interval (95% CrI) and prediction interval (95% PI) using the *bayesmeta* R package (Version 4.3.2, R Foundation for Statistical Computing, Vienna, Austria).^13^ The overall treatment effect (μ) was assigned a weakly informative prior μ∼Normal(0, 0.71^2^) on the log-OR scale, implying that 95% of plausible ORs lie between approximately 0.25 and 4, which reflects empirically observed ranges of treatment effects in RCTs.^14^ Likewise, an evidence-based prior based on published meta-analyses was used for τ.^15^ The probability of any benefit (OR < 1) and a number needed to treat (NNT) < 20 (5% absolute risk difference) were calculated from the posterior and predictive distributions. The latter provides a more conservative estimation of these probabilities in future RCTs.

## Results

### Search results and study characteristics

A total of 130 unique entries were screened and 2 were included in the review^6,7^ (Figure S1, available as Supplementary data at *JAC-AMR* Online). The two RCTs comprised 102 patients: 53 were randomized to prophylaxis and 49 to placebo. Both trials recruited patients re-exposed to systemic antibiotics within 180 days of their preceding episode of CDI and were stopped early. Collectively, only 40.5% of the target sample size was attained. San-Juan *et al.* used a fixed 10-day duration of vancomycin 125 mg PO QID,^6^ whereas Keating *et al.* used 125 mg PO daily for the duration of systemic antibiotics plus 5 days.^7^ A total of 55.1% and 35.8% assigned to the placebo and vancomycin groups, respectively, experienced rCDI at 56 days. Additional study details are listed in Table 1.

**Table 1.** Study characteristics

Author, year	Country	Years of recruitment	Design	Permitted timeframe from CDI to recruitment	Definition of rCDI	Inclusion criteria	Exclusion criteria	Vancomycin regimen	Planned sample size	Recruited Sample Size
San-Juan, 2026^6^	Spain	2022–24	Double-blind	180 days	≥3 new unformed stools in <24 h (or >200 mL in patients with a colostomy) and detection of toxigenic *C. difficile* via either toxin EIA or PCR	Hospitalized adults with CDI within the preceding 180 days and 24–72 h of full-dose systemic antibiotics	Chronic diarrheal diseases, CDI diagnosis within 3 days, exposure to vancomycin, or other CDI-active drug for >48 h in the preceding 3 days	Vancomycin 125 mg PO q6h × 10 days	102	21
Keating, 2025^7^	USA	2018–23	Double-blind	180 days	≥3 unformed stools in <24 h and a positive toxin EIA or reflex CCNA (if the toxin EIA was negative)	Adults with one or more episodes of CDI within the preceding 180 days, completion CDI treatment, and receipt of systemic antibiotics for <2 weeks	>72 h of systemic antibiotics, contraindication to the study drugs, current use of oral vancomycin, metronidazole, or tetracycline monotherapy, suspected CDI or other active gastrointestinal conditions, gastrointestinal surgery, total colectomy, bariatric surgery	Vancomycin 125 mg PO daily for the duration of systemic antibiotics + 5 days	150	81

### Study quality

Both RCTs were considered at low risk of bias (Figure S2).

### Meta-analysis

**Figure 1.**
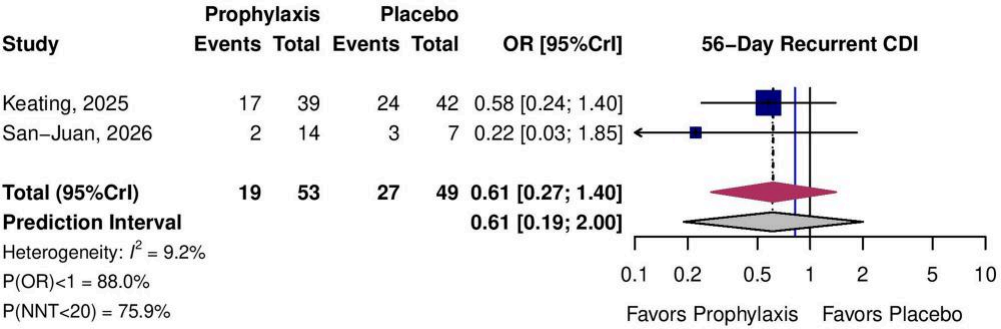
Forest plot of 56-day rCDI. The blue vertical line corresponds to OR = 0.82, which corresponds to an NNT < 20.

The pooled OR for 56-day rCDI was 0.61 (95% CrI = 0.27–1.40), favouring vancomycin prophylaxis (Figure 1). This corresponded to an 88.0% posterior probability of any benefit and a 75.9% probability that the NNT was <20. The 95% PI was 0.19–2.00 and, using the predictive distribution, the probabilities of any benefit and NNT < 20 were 82.9% and 72.0%, respectively.

### Certainty of evidence

The certainty of evidence that secondary vancomycin prophylaxis reduces rCDI in patients re-exposed to systemic antibiotics was rated as low, with downgrading due to the width of the 95% PI.

## Discussion

Using RCT estimates, this study suggests a moderate probability of any benefit for secondary vancomycin prophylaxis. Nevertheless, there remains significant equipoise for future RCTs because the 95% PI was wide, the data are limited to 102 patients, both studies closed early, data on delayed rCDI past 56 days were sparse, and the event rates were unexpectedly high across both trials.^2^

We will explore leveraging the 95% PI from this meta-analysis as the Bayesian prior for our ongoing adaptive RCT of secondary vancomycin prophylaxis (SPORES-V, NCT06979609).^16^ Several other trials of vancomycin prophylaxis are closed or underway (Table S1). Challenges of conducting an RCT in this space include recruitment difficulties that were experienced by the prior RCTs, as well prevalent off-label vancomycin prophylaxis in certain settings.^5,17^

Future RCTs should explore rCDI beyond 56 days because the relative efficacy of vancomycin prophylaxis appeared to wane during this timeframe in the Keating *et al.* trial,^7^ which could suggest that rCDI was being delayed and not truly prevented. The San Juan *et al.*^6^ trial did not provide follow-up data beyond 56 days, precluding meta-analysis.

The Keating *et al.* trial suggested more prolonged VRE colonization in the vancomycin group.^7^ VRE colonization was not reported by San Juan *et al.*^6^ and, therefore, could not be meta-analysed. Although prolongation of VRE colonization is concerning, if vancomycin prophylaxis is efficacious in the prevention of rCDI, this benefit may outweigh theoretical microbiome harms.^18^ Further, a reduction in rCDI could itself prevent or reduce hospitalization, which in and of itself can lead to downstream colonization with multidrug-resistant organisms. Reassuringly, a large observational study of patients with CDI did not find an increased risk of VRE infection with vancomycin versus metronidazole, although colonization was not assessed.^19^ Nevertheless, VRE colonization should be measured by future trials.

Strengths of this study include the Bayesian design that enables a more nuanced, probabilistic view of efficacy and the sole inclusion of RCTs. The predominant limitation was the small sample size, limiting power. Additionally, differences in the vancomycin prophylaxis regimens used (125 mg daily versus 125 mg QID) may have introduced heterogeneity into the estimate of efficacy and could theoretically have differential impacts on VRE colonization.

It is looking increasingly likely that vancomycin secondary prophylaxis may be effective in the short term. However, adequately powered multicentred RCTs are still required in this space before this practice becomes universally implemented.

## Supplementary Material

dlag141_Supplementary_Data

## Supplementary data

Figures S1 and S2 and Table S1 are available as Supplementary data at *JAC-AMR* Online.
