# Supplementary material for: Secondary vancomycin prophylaxis during antibiotic re-exposure for the prevention of recurrent Clostridioides difficile infections: a systematic review and Bayesian meta-analysis
Source: JAC Antimicrob Resist. 2026 Jul 23;8(4):dlag141. doi: 10.1093/jacamr/dlag141 (PMC13392467; doi:10.1093/jacamr/dlag141)
Supplement: dlag141_Supplementary_Data [file dlag141_supplementary_data.docx]

**SUPPLEMENTAL FIGURES**

**Figure S1.** PRISMA diagram.


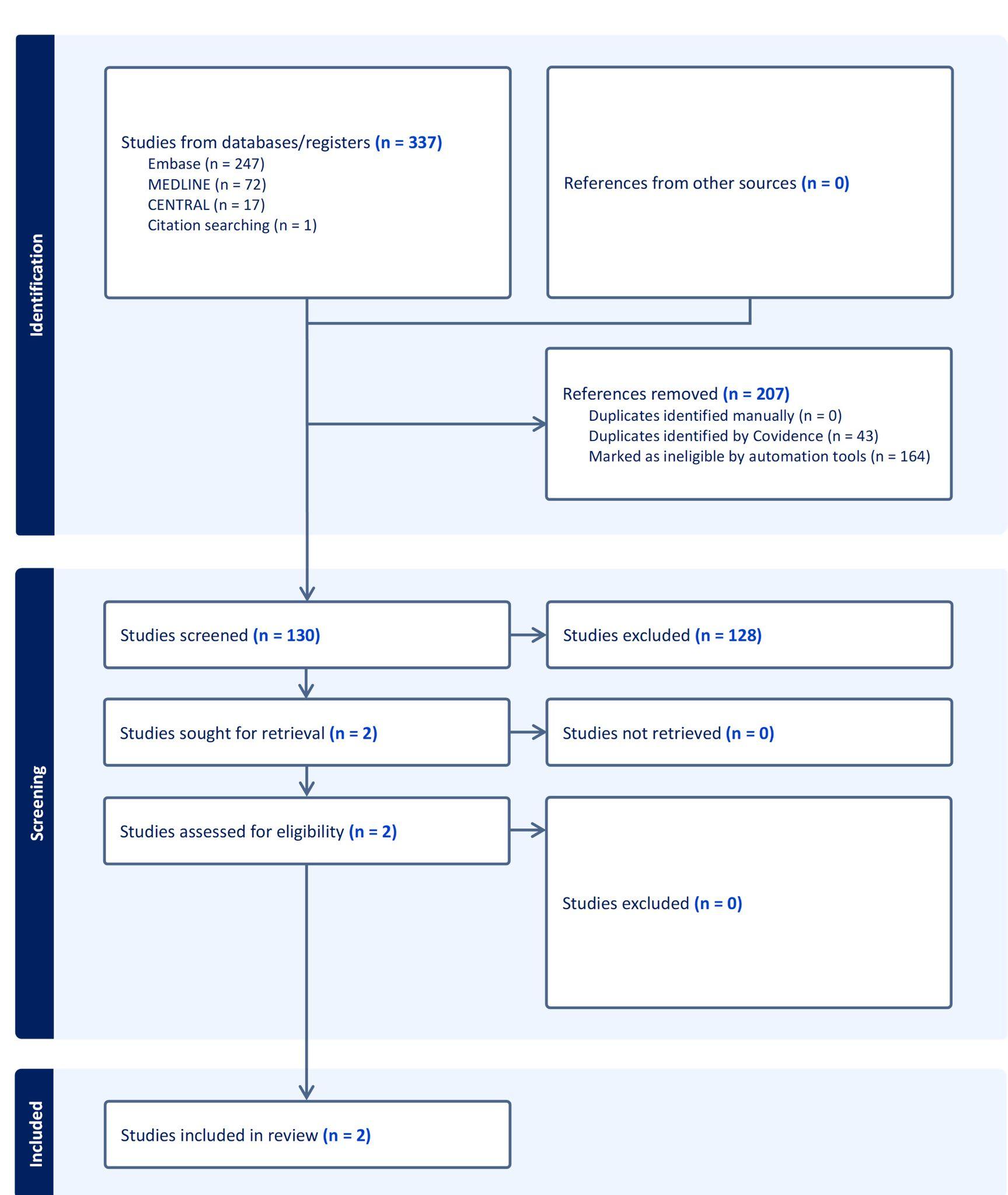


**Figure S2.** Cochrane RoB2 risk of bias assessment.


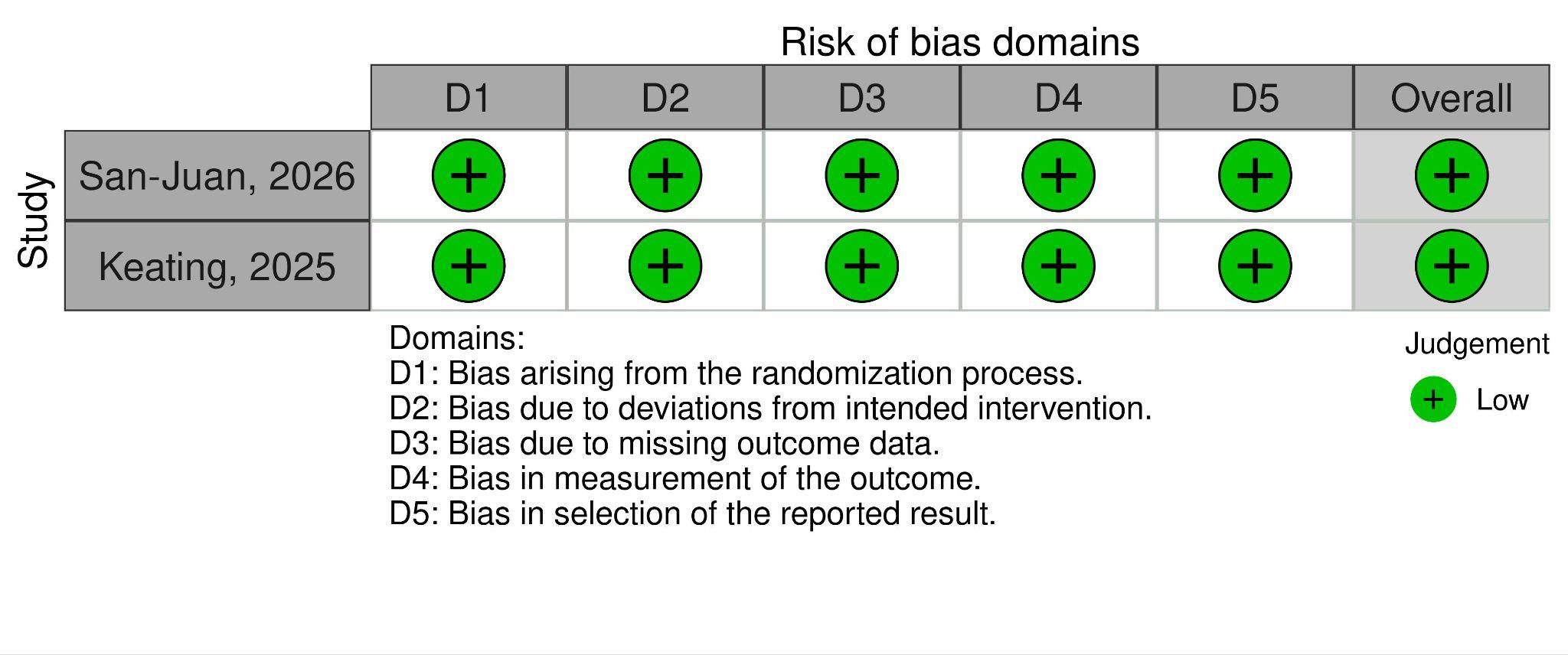


**SUPPLEMENTAL TABLES**

**Table S1.** Unpublished RCTs on secondary vancomycin prophylaxis during antibiotic re-exposure listed on [clinicaltrials.gov](http://clinicaltrials.gov) from inception to June 18, 2026 by searching *Clostridium difficile* and vancomycin.

| **NCT** | **Country** | **Planned/Actual Years of Recruitment** | **Regimens Compared** | **Planned/Actual Sample Size** | **Status** |
| --- | --- | --- | --- | --- | --- |
| NCT03466502 | USA | 2018-2022 | Vancomycin 125mg PO BID vs. vancomycin 125mg PO daily vs. no intervention | 104 | Completed |
| NCT03200093 | USA | 2017-2021 | Vancomycin 125mg PO daily for duration of antibiotics +3 days or if the duration of antibiotics is >14 days then for the duration of antibiotics +7 days vs. placebo | 65 | Completed |
| NCT04000555 | USA | 2019-2022 | Vancomycin 125mg PO BID for the duration of antibiotics vs. placebo. | 26 | Terminated early |
| NCT02237859 | USA | 2014-2016 | Vancomycin 125mg PO daily for the duration of antibiotics vs. placebo | 140 | Unknown |
| NCT06979609 | Canada | 2025-2029 | Vancomycin 125mg PO BID for the duration of antibiotics + 7 days vs. placebo | 300 | Recruiting |
